# MiR-486-5p inhibits IL-22-induced epithelial-mesenchymal transition of breast cancer cell by repressing Dock1

**DOI:** 10.7150/jca.30596

**Published:** 2019-08-19

**Authors:** Hongli Li, Qingjie Mou, Peirui Li, Zhiyi Yang, Zhaoyan Wang, Jie Niu, Yuanyuan Liu, Zhiliang Sun, Shijun Lv, Baogang Zhang, Chonggao Yin

**Affiliations:** 1 Medicine Research Center, Weifang Medical University, Weifang, China.; 2 Department of Oncology, Clinical Medical College, Weifang Medical University, Weifang, China; 3 Department of Thyroid and Breast Surgery, Affiliated Hospital of Weifang Medical University, Weifang, China; 4 Department of Pathology, Clinical Medical College, Weifang Medical University, Weifang, China.; 5 College of Nursing, Weifang Medical University, Weifang, China.; 6 College of Biological Science and Technology, Weifang Medical University, Weifang, China.

**Keywords:** miR-486-5p, epithelial-mesenchymal transition, Dock1, breast cancer, IL-22

## Abstract

Epithelial-mesenchymal transition (EMT) is one of important steps that lead to cancer metastasis. Interleukin-22 (IL-22) is a T helper 17 (Th17) cells-secreted cytokine, it can promote invasion and metastasis of many cancers. MiR-486-5p is a microRNA that known to function as a tumor suppressor, and bioinformatics analysis predicts that Dock-1 has a binding site of miR-486-5p. In current research, we examined the relative expression levels of miR-486-5p and Dock-1 in 80 pairs of breast cancer tissues and corresponding adjacent normal tissues, also the effects of modifying their levels in cultured cells. We illustrated that IL-22 and Dock1 promote the invasion, metastasis, and EMT of breast cancer using Transwell invasion assay, western blot and immunofluorescence. MiR-486-5p directly bound the Dock1 mRNA 3' untranslated region and inhibited IL-22-induced EMT of breast cancer cells via the Dock1/NF-κB/Snail signaling pathway. Dock1 overexpression reversed the effect caused by the overexpression of miR-486-5p. Overexpression of miR-486-5p or downregulation of Dock1 reduced pulmonary metastasis in mice. This study provided insight into a potential mechanism where miRNAs regulate breast cancer metastasis and provided a novel therapeutic target for breast cancer treatment.

## Introduction

Breast cancer is a common female cancer but is usually not fatal when diagnosed in its early stage. According to the 2015 WHO World Cancer Report there are about 14 million new patients and 8.2 million deaths of cancer worldwide 1. Despite the progress in clinical treatment strategies, its mortality rate remains extremely high. Metastasis is responsible for the majority of deaths that caused by breast cancer. Particular transcription factors modulate epithelial- mesenchymal transition (EMT), which is the mechanism of metastasis that responsible for cancer-related death.

Interleukin-22 (IL-22) is a Th17-secreted cytokine, it can bind to a class II cytokine receptor that contains IL-22R1 and IL-10R2 and also influence a variety of immune reactions 2. IL-22 also promotes the metastasis and invasion of cancer cells 3, 4. Dedicator of cytokinesis 1 (DOCK1), also called DOCK180, is a typical one of evolutionarily conserved DOCK superfamily. DOCK1 influences various cellular processes, such as polarity, phagocytosis, clearance of apoptotic cells, motility, and invasion through regulating the activation of GTPaseRac 5. Migration and invasion of many cancer cells were thought to be related with upregulation of DOCK1 6-9.

MicroRNAs (miRNAs), a class of endogenous, evolutionarily conserved small non-coding RNA molecules, can modulate posttranscriptional processing of target genes and lead to degradation of target mRNAs and their translational repression 10. Several researches manifested that miRNAs can act as oncogenes or anti-oncogenes. MiR-183 and miR-494 are two miRNAs that serve critical functions in breast cancer metastasis. miR-183 mediates RB1 protein to suppress MDA-MB-231 cells 11. MiR-152 represses the ROCK1 protein, therefore inhibits cell proliferation and migration in breast cancer 12. MiR-143 downregulates the levels of cancer-related factors, leading to the inhibition of metastasis and proliferation of MDA-468 cells 13. MiR-486-5p, an anti-oncogene in many tumors, might be estimate tumor marker for evaluating prognosis in patients with esophagus cancers and gastric carcinomas 14. MiR-486-5p repressing PIM-1 suppresses cell proliferation in human breast cancer cells 15. In current study, we determined the role of miR-486-5p, Dock1, and IL-22 in breast cancer EMT.

## Materials and Methods

### Cell lines and Plasmids

In order to investigate the effect in diverse cell lines, the high invasive human breast cancer cells MDA-MB-231 and MDA-MB-453, low invasive T47D and MCF-7 were selected for this study. All cell lines were obtained from ATCC. MCF-7 cells were incubated in MEM with 10% FBS and 1% sodium pyruvate. MDA-MB-231, T47D, and MDA-MB-453 cells were incubated in DMEM and RPMI1640 that contained 10% FBS, at 37°C atmosphere of 5% CO_2_.

MiR-486-5p, the vector, Anti-miR-486-5p, anti- control, Dock1 RNAi vector, and Dock1 overexpression vector were obtained from GeneChem (Shanghai, China). Wildtype or mutant 3'-untranslated regions (3'-UTR) of Dock1, potential miR-486-5p target sites were amplified and subcloned into pGL3 vector.

### Patients and Breast cancer tissues

80 pairs of frozen (liquid nitrogen) breast cancer tissues and adjacent normal tissues (ANTs) were acquired from patients with breast cancer at the Affiliated Hospital of Weifang Medical University from 2012 to 2014. In this study, ANTs means the adjacent tissues that no breast cancer cells can be detected by microscope. To ensure that ANTs do not contain tumor cells, a paraffin-embedded block was routinely taken from each ANTs before frozen, the blocks with identified tumor cells were excluded. None of the participates have had radiotherapy or chemotherapy before surgery. All patients gave their informed consent for the use of tissues and the hospital ethics committee approved the study.

### Invasion Assays

The transwell invasion system consisted of fluid-filled stacked compartments that separated by a Matrigel coated porous membrane filter. We employed reconstituted basement membrane matrigel (BD, San Jose, Biosciences, MA, USA) as the substrate for invasion, to evaluate the impact of IL-22, Dock1, and miR-486-5p on the invasion of breast cancer cells as previously described 16. In brief, we incubated all cells at 37 ℃ for 2 days, then placed onto the upper wells (8 μm pore size, 24-well insert) using a culture medium without serum. Added complete medium into bottom wells that contained 10% FBS or IL-22 as chemoattractant. After 24 h, the remaining cells in the upper wells (non-invading cells) were removed with a cotton swab. And then the cells that invaded through the matrigel were colorated with 10% Giemsa stain. The number of invading cells were calculated using light microscope (Olympus, Tokyo, Japan).

### Western blot

Western blot analyses were conducted as reported 17. The antibodies used were: anti-Dock1 (abcam, 1:1000), Vimentin (abcam, 1:1000), E-cadherin (abcam, 1:1000), Snail (abcam, 1:500), p-IκB (CST, 1:1000), IκB (CST, 1:1000) antibody. All experiments were repeated triply. The gray value of western blot were evaluated and quantified using ImageJ software.

### Quantitative real-time PCR analysis (qRT-PCR)

Stem-loop qRT-PCR was conducted to determine the relative expression of miR-486-5p. The stem-loop RT-PCR method was designed to detect and quantify miRNAs in a fast, specific, accurate and reliable way. First, a miRNA-specific stem-loop RT primer is hybridized to the miRNA and then reverse transcribed. Next, the reverse transcribed product is amplified and monitored in real time. U6 levels were used for normalization. Trizol was used to extract total RNA from all cells and fresh breast cancer tissues. The concentration was quantified using a Nano Drop Spectrophotometer (NanoDrop Technologies, USA). CDNA was obtained using the MMLV Reverse Transcription Kit (Promega, USA) with 5 ng of total RNA. QRT-PCR was conducted by use of an Applied Biosystems 7500 Sequence Detection system (Thermo Fisher Scientific, USA). The quantity of miRNA of corresponding reference gene could be computed using 2^-∆∆CT^ method, where ∆CT= (CT_miRNA_ - CT _reference RNA_). Comparison of miRNA expression was based on a comparative CT method(∆∆CT), and the relative miRNA expression can be normalized using 2^-∆∆CT^ method, where ∆∆CT=(CT_miRNA_ - CT_reference RNA_) - (CT_calibrator_ - CT_reference RNA_) 18. Sequences of primers were as follows, miRNA-486-5P RT primer: 5'-GTCGTATCCAGTGCAGGGTCCGAGGTATTCGCACTGGATACGACCTCGGG-3', Forward primer: 5'-CGCGTCCTGTACTGAGCTGC-3', Reverse primer: 5'-ATCCAGTGCAGGGTCCGAGG-3'. MiRNA-506 RT primer: 5'-GTCGTATCCAGTGCAGGGTCCGAGGTATTCGCACTGGATACGACTTAAGT-3', Forward primer: 5'-GCGCGTATTCAGGAAGGTGTT-3', Reverse primer: 5'-AGTGCAGGGTCCGAGGTATT-3'.

### Luciferase reporter assay

The wildtype full-length 3'UTR fragment of the Dock1 gene was obtained using PCR from human genomic DNA. The predicted seed sequences were mutated from the 3'UTR of Dock1 using the overlapping PCR method. PCR products were cloned into the pGL3 vector (Promega, Madison, USA). For luciferase reporter assay, the MDA-MB-231 and MCF-7 cells were incubated in 24-well plates, 100 ng of pGL3-Dock1-3'-UTR-WT or pGL3-Dock1-3'-UTR-MUT and indicated miRNA vector were co-transfected to MDA-MB-231 and MCF-7 cells respectively using Lipofectamine 2000. After 48h, the cells were obtained and the activity of firefly and Renilla luciferase were measured by Dual-Luciferase Reporter Assay System (Promega, Madison, USA), then normalized to those of Renilla luciferase activity.

### Animal studies

The mice were maintained at the animal experiment center of Weifang Medical University under defined conditions. All procedures were approved by the animal care and use committee of the institution and conformed to legal mandates and national guidelines for the care and maintenance of laboratory animals. 4-weeks-old female SCID mice were used. Scr/MDA-MB-231 or SiDock1/MDA-MB-231 cells and NC /MDA-MB-231 or miR-486-5p/MDA-MB-231 cells were injected into mammary fat pads of SCID mice (n = 10 in each group). When the xenografts were palpable (~0.5 cm in diameter), 100 ng/kg IL-22 was injected intratumorally twice a week for 4 consecutive weeks. After 8 weeks, the mice were sacrificed and the tumors were harvested. Western blot was used to assess the levels of Dock1, E-cadherin, snail, and Vimentin. The lung tissues were fixed and embedded in paraffin, and then serial sections, hematoxylin and eosin staining were implemented to detect lung metastasis.

### Statistical analysis

All analyses were performed by use of SPSS 22.0 statistical software package. Results were presented as mean ± standard deviation (s.d.). Statistical significance for comparisons between groups was evaluated by use of analysis of variance (ANOVA). The chi-square test was implied to explore the relationships among miR-486-5p, Dock1 level, and clinicopathological characteristics. *P* < 0.05 was considered with statistical significance.

## Results

### IL-22 faciliated invasion and EMT in breast cancer

**Figure 1:**
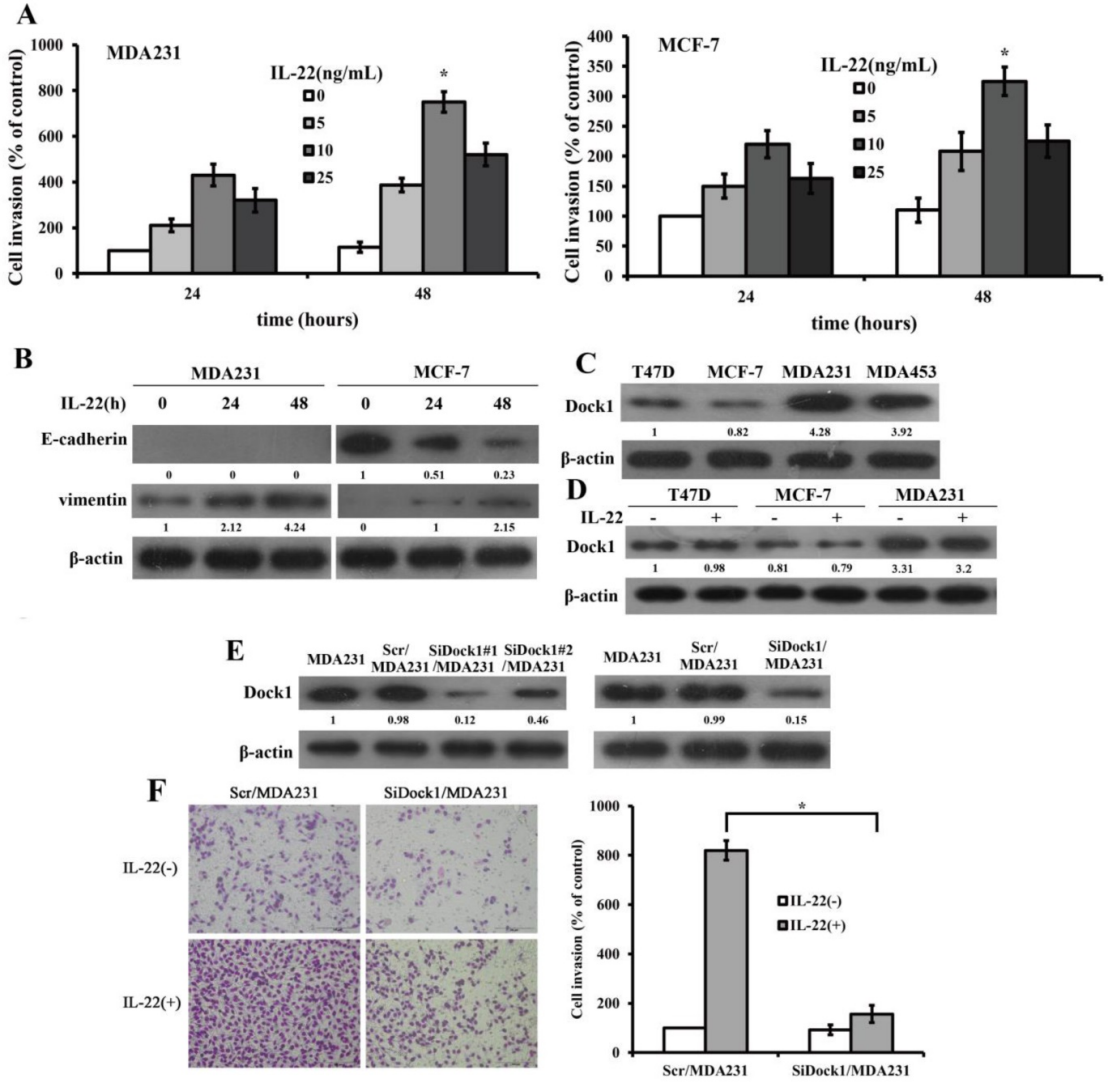
IL-22 promoted invasion and EMT of breast cancer cells. Dock1 was required for IL-22-mediated MDA-MB-231 cells invasion and EMT.** A.** The IL-22-triggered invasion of breast cancer cells was analyzed. Left, quantitative analysis of MDA-MB-231 cells under diverse concentrations of IL-22 (0-25 ng/mL) with time gradience. Right, quantitative analysis of MCF-7 cells under diverse concentrations of IL-22 (0-25 ng/mL) with time gradience. *P< 0.05. **B.** Level of E-cadherin and Vimentin in MDA-MB-231 and MCF-7 cells with and without 10 ng/mL IL-22 was examined by western blot at different time points. **C.** Expression of Dock1 protein in cultured breast cancer cells. **D.** Levels of Dock1 protein in breast cancer cells were assessed by western blot after 48 hours with and without stimulation of 10 ng/mL IL-22. **E.** Western blot analysis of Dock1 expression in indicated cells. **F.** The IL-22-induced invasion of MDA-MB-231 cells was evaluated. Left, photographs of invading cells (magnification, 200×). Right, quantification of invading cells. *P < 0.05. For **B, C, D, E,** data of western blot were representative of triply repeated experiments. Used β-actin as loading control. Quantifications of relative protein level were shown below the blots.

To determine the impact of IL-22 on invasion of breast cancer cells, we conducted a Transwell invasion assay. Transwell invasion assay results manifested that the invasion rate of MDA-MB-231 and MCF-7 were highest when stimulated with 10 ng/mL IL-22 (Figure 1A).To investigate whether IL-22 facilitated breast cancer cells invasion by increasing EMT, we assessed the levels of E-cadherin and Vimentin with and without 10 ng/mL IL-22 stimulation with time gradient. IL-22 stimulation increased the expression of Vimentin in MDA-MB-231 and MCF-7 cells but decreased the level of E-cadherin in MCF-7 cells (Figure 1B). To determine whether Dock1 acted a critical part in IL-22-trigered EMT, we detected the expression of Dock1 with and without IL-22 stimulation in different cell lines. We found higher levels of Dock1 in high invasive cells MDA-MB-231 and MDA-MB-453, but lower levels in low invasive cells MCF-7 and T47D (Figure 1C). IL-22 did not affect Dock1 protein levels in breast cancer cells (Figure 1D). Subsequently, we chose the least invasive cell line MCF-7 and the most invasive cell line MDA-MB-231 to investigate the molecular mechanism *in vitro*.

### Dock1 was required for IL-22-induced cell invasion and EMT

To determine the participation of Dock1 in the IL-22-trigered invasion of MDA-MB-231 cells, we constructed specific siRNA plasmid of Dock1 to knockdown Dock1 in MDA-MB-231 cells. We also constructed control plasmid to transfect MDA-MB-231 cells (Scr/MDA-MB-231). We obtained the stable Dock1 knockdown MDA-MB-231 cells (SiDock1/MDA-MB-231) using SiDock1#1 sequence (Figure 1E). To identify the roles of Dock1 in IL-22-induced breast cancer cells invasion, we conducted a Transwell invasion assay. The invaded cell number of SiDock1/MDA-MB-231 was considerably fewer than Scr/MDA-MB-231 cells under stimulation of 10 ng/mL IL-22. (Figure 1F).

**Figure 2:**
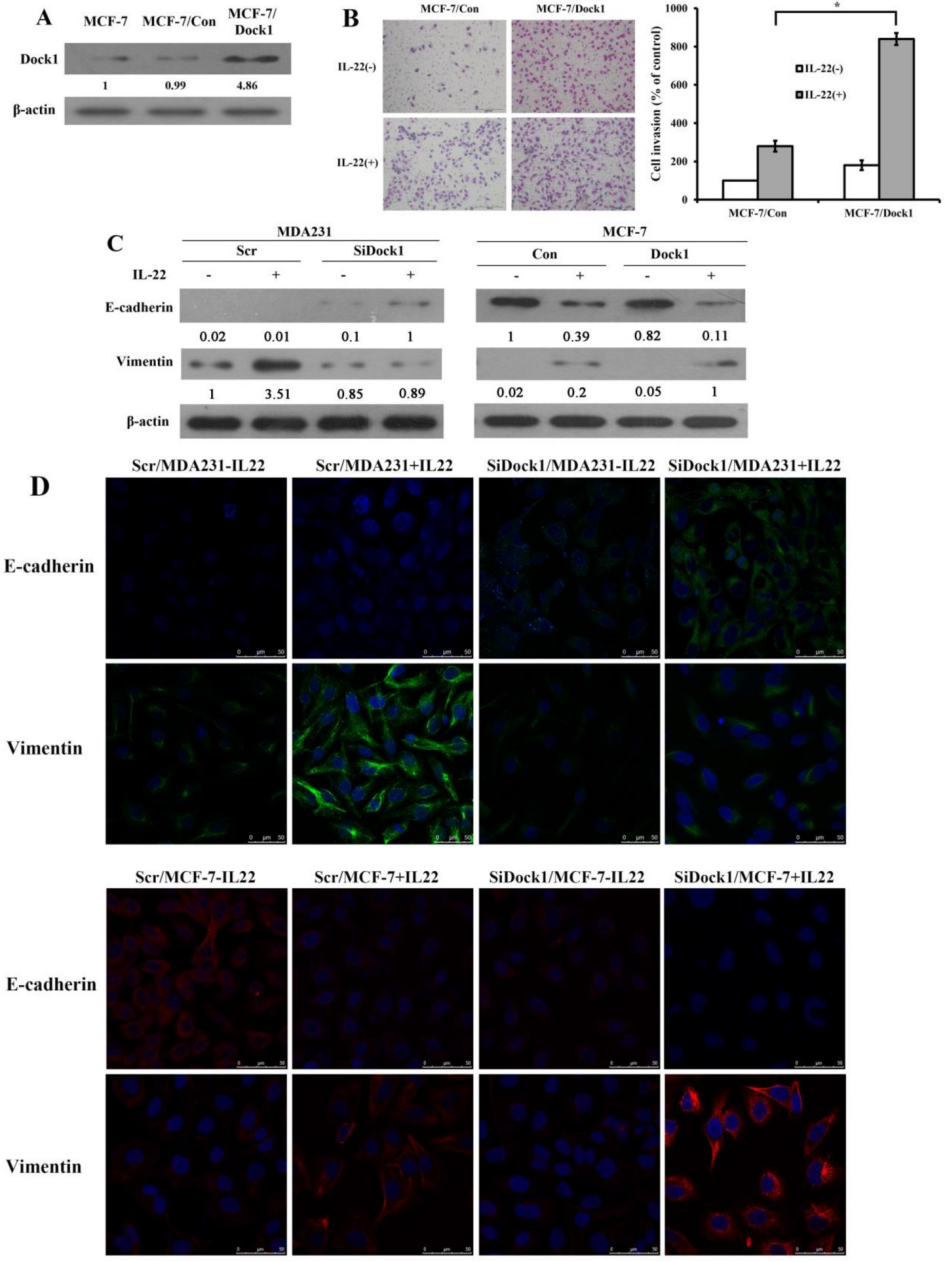
Dock1 was required for IL-22-mediated MCF-7 cell invasion, metastasis, and EMT.** A.** Western blot analysis of Dock1 expression in indicated cells. **B.** The IL-22-induced invasion of MCF-7 cells was assessed. Left, photographs of invading cells (magnification, 200×). Right, quantification of invading cells. *P< 0.05. The results shown were representative of triply repeated experiments. **C.** Western blot of E-cadherin level and Vimentin level in indicated cells after 48 h with or without 10 ng/mL IL-22. **D.** Fluorescence microscopy of stained E-cadherin and Vimentin presented in indicated cells after 48h with IL-22. Nuclear DNA was stained with DAPI (blue). Scale bar: 50 μm. For **A** and** C**, western blot results were representative from triply repeated experiments. Used β-actin as loading control. Quantifications of relative protein level are shown below the blots.

In the meantime, we used GV230-Dock1 plasmid to construct and select stably transfected cell clones. All stably transfected clones had similar phenotypes. We selected the optimal clone 2, entitled as Dock1/MCF-7cells. The GV230 vector was used to generate the vector control cells, Con/MCF-7. The protein expression of Dock1 stable clones was detected by western blot analysis (Figure 2A). The invaded cell number of Dock1/MCF-7 was prominently higher than Con/MCF-7 under stimulation of 10 ng/mL IL-22. (Figure 2B). To investigate whether Dock1 involves in IL-22 triggered breast cancer invasion by EMT, EMT markers (E-cadherin and Vimentin) were assessed using western blot and immunofluorescence with or without 10 ng/mL IL-22 stimulation for 48 hours. Vimentin expression was reduced and E-cadherin expression was upregulated in SiDock1/MDA-MB-231 cells than in Scr/MDA-MB-231 cells with 10 ng/mL IL-22 stimulation after 48 hours (Figure 2C, left). In Dock1/MCF-7 cells, the expression of E-cadherin was downregulated, while Vimentin expression was upregulated (Figure 2C, right). The same results were observed in immunofluorescence (Figure 2D). Hence, the results manifested that Dock1 increased mesenchymal properties of breast cancer cells and triggered EMT under stimulation of IL-22.

### MiR-486-5p directly targets and represses Dock1

**Figure 3:**
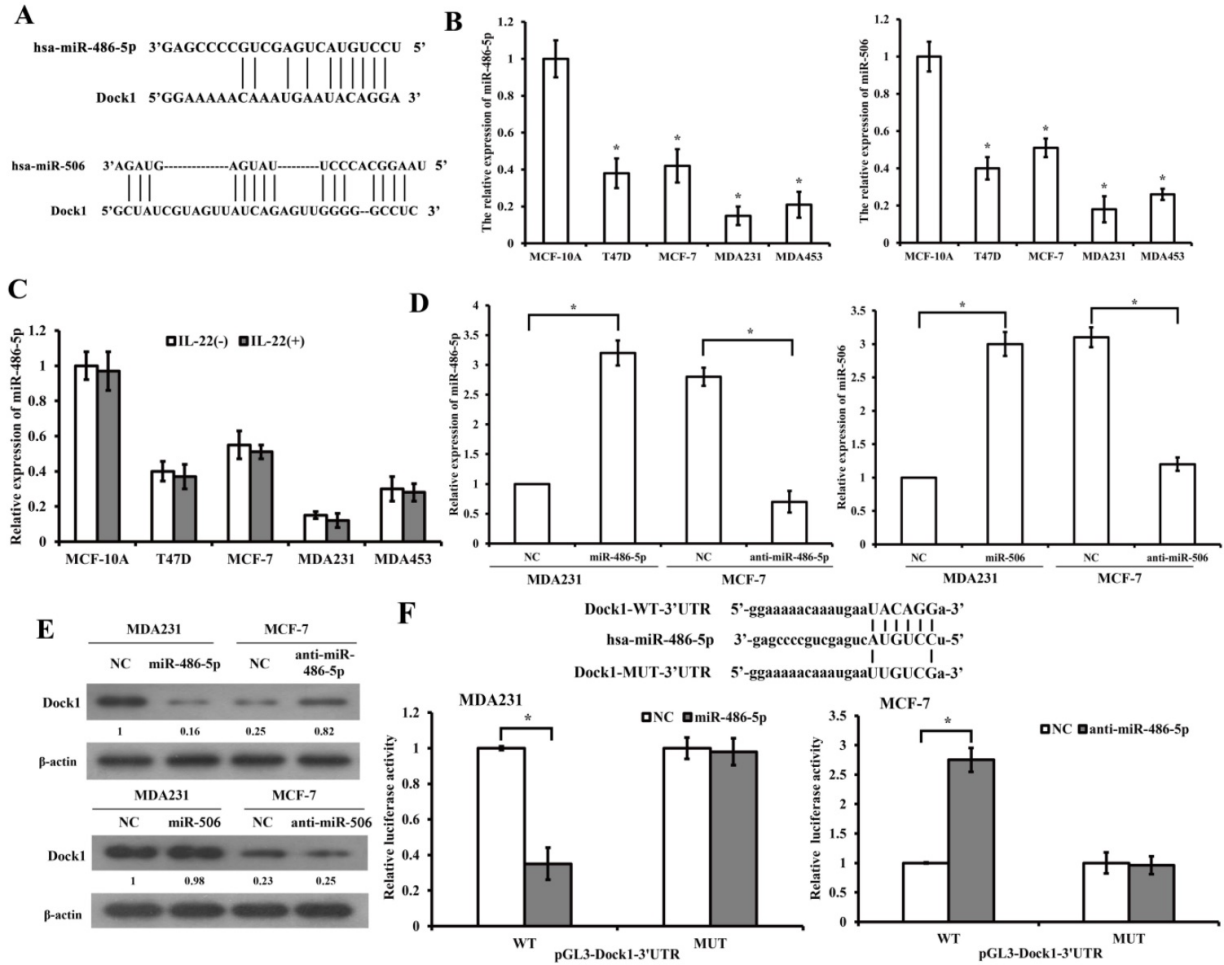
Dock1 was mediated by miR-486-5p.** A.** Predicted target sites of miR-486-5p and miR-506 to 3'-UTRs of Dock1. **B.** QRT-PCR analysis of miR-486-5p and miR-506 in MCF-10A cells and human breast cancer cells. **C.** MiR-486-5p levels in MCF-10A cells and breast cancer cells with or without 10 ng/mL IL-22 were determined by qRT-PCR. **D.** Relative expression of miR-486-5p and miR-506 in indicated cells. **E.** Western blot analysis of Dock1 protein expression in indicated cells. Used β-actin as loading control. Quantifications of relative protein level are shown below the blots. **F.** The relative luciferase activity was tested in MDA-MB-231 and MCF-7 cells after co-transfected with pGL3-WT or MUT Dock1 3' UTR and NC, miR-486-5p or anti-miR-486-5p. For **B, C, D,** and **F**, *P<0.05.

We predicted relevant miRNAs that modulate Dock1 using miRNA.org (http://www.microrna.org/microrna/searchGenes.do) and Targetscan (http://www.targetscan.org/). The databases revealed that miR-486-5p and miR-506 are potential miRNAs that target Dock1 (Figure 3A). QRT-PCR demonstrated that miR-486-5p and miR-506 were lower in four human breast cancer cell lines compared with MCF-10A cells (Figure 3B). Additionally, the results demonstrated that IL-22 did not influence the level of miR-486-5p in breast cancer cells (Figure 3C). The miRNA plasmid and negative control (NC) plasmid were synthesized and transfected into MDA-MB-231 and MCF-7 cells (Figure 3D). Western blot illustrated that miR-486-5p suppressed the level of Dock1 in transfected cells compared with NC (Figure 3E). However, the increase of miR-506 in MDA-MB-231 cells and the decrease of miR-506 in MCF-7, did not inhibit the expression of Dock1. To confirm whether the 3'-UTR of Dock1 is a main acting site of miR-486-5p in this regulation, we designed wildtype and mutant Dock1 3'-UTR downstream of luciferase reporter genes. Wildtype or mutant Dock1 vector was co-transfected into MDA-MB-231 cells with miR-486-5p or negative control. The luciferase activity of MDA-MB-231 cells after co-transfection with wildtype Dock1 3'-UTR and miR-486-5p vector was significantly reduced compared with corresponding controls. The opposite results were obtained in MCF-7 cells that co-transfected with wildtype Dock1 3'-UTR and anti-miR-486-5p (Figure 3F), indicating that 3'-UTR of Dock1 mRNA is a direct binding site of miR-486-5p. All above, the results manifested that miR-486-5p downregulated Dock1 expression through binding its 3'-UTR.

### MiR-486-5p inhibits IL-22-triggred EMT via Dock1/NF-κB/Snail pathway

**Figure 4:**
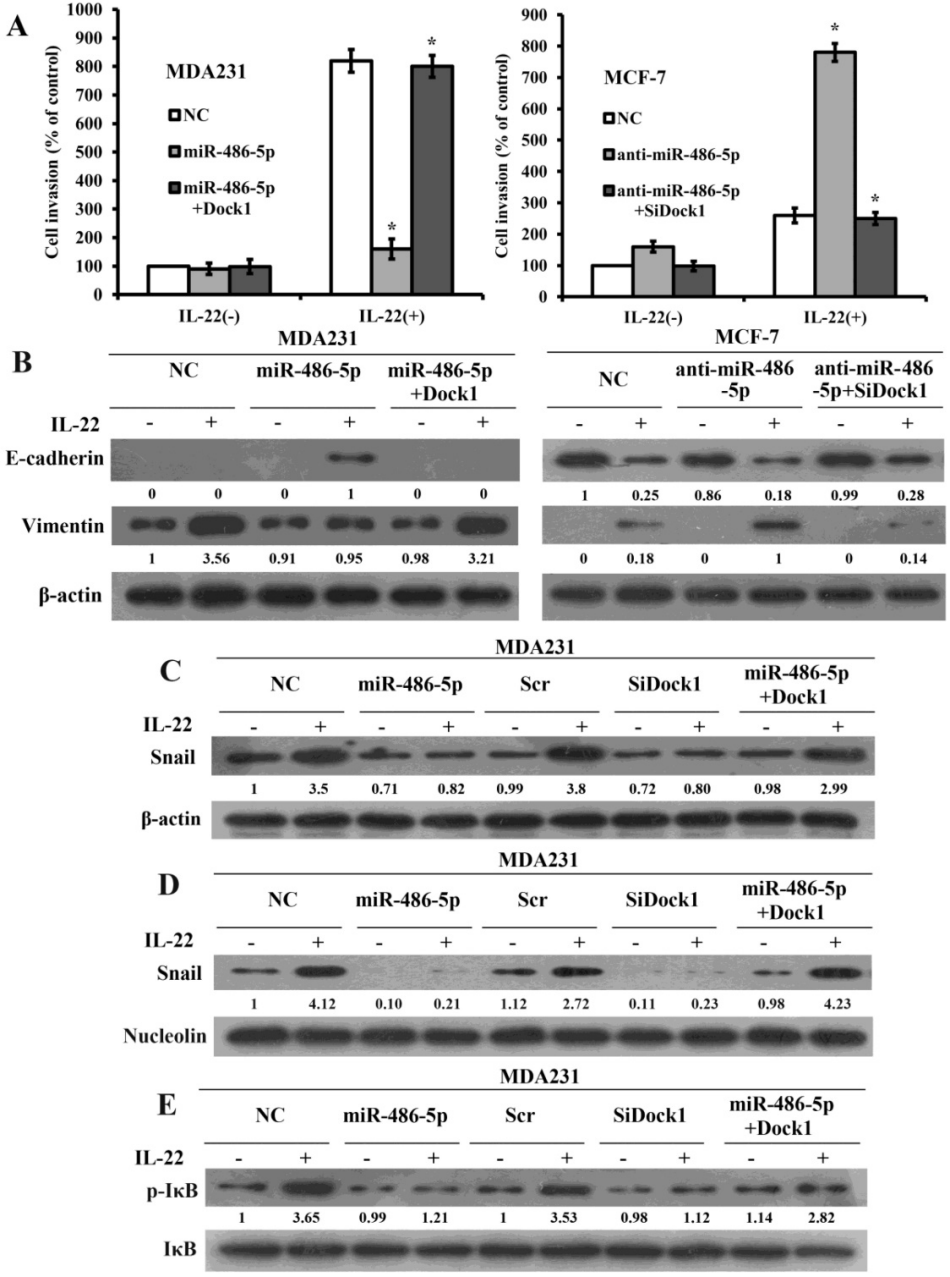
MiR-486-5p inhibits IL-22-induced EMT through the Dock1/ NF-κB/Snail signaling pathway.** A.** Transwell invasion assays of MDA-MB-231 and MCF-7 cells were conducted after transfection with NC, miR-486-5p, miR-486-5p+Dock1, anti-miR-486-5p, and anti-miR-486-5p+SiDock1 as indicated. *P<0.05. **B.** Western blot of E-cadherin and Vimentin levels in indicated cells after 48 hours with or without 10 ng/mL IL-22. Used β-actin as a loading control. **C.** Expression of Snail was tested by western blot in indicated cells after 48 hours with or without 10 ng/mL IL-22. Used β-actin as loading control. **D.** Nuclear level of Snail was evaluated by western blot in indicated cells after 48 hours with or without 10 ng/mL IL-22. Used Nucleolin as loading control. **E.** Western blot of IκBα expression and phosphorylation status in the indicated cells. Used IκBα as loading control. IL-22, 10 ng/mL. Data were presented from representative of three or more independent experiments. Quantifications of relative protein levels are shown below the blots.

To illuminate the significance of miR-486-5p-modulated inhibition of Dock1 on cell invasion and EMT, we expressed open reading frames (ORFs, without their respective 3-UTRs) of Dock1 in miR-486-5p-overexpressed cells and silenced Dock1 in miR-486-5p-silenced cells, then tested the invasion by transwell assay. As expected, overexpression of Dock1 considerably rescued the invasion that reduced by miR-486-5p, and knockout of Dock1 prominently reversed the invasion that increased by anti-miR-486-5p (Figure 4A), suggesting that the inhibiting effect of miR-486-5p on breast cancer cells is mainly carried out by downregulation of Dock1. At the same time, we detected EMT markers in all cells by use of western blot after 48 hours with or without stimulation of 10 ng/mL IL-22. The level of Vimentin was reduced, whereas the level of E-cadherin was increased in miR-486-5p/MDA-MB-231 cells after 48 hours with or without stimulation of 10 ng/mL IL-22 compared with NC/MDA-MB-231 cells. Re-expression of Dock1 completely inhibited miR-486-5p-mediated upregulation of E-cadherin and promoted miR-486-5p-mediated downregulation of Vimentin (Figure 4B, left). In anti-miR-486-5p/MCF-7 cells, E-cadherin expression was downregulated, but Vimentin expression was upregulated. Overexpression of Dock1 rescued miR-486-5p-mediated alteration of E-cadherin and Vimentin simultaneously. (Figure 4B, right). The attenuation of E-cadherin is a feature of EMT. The transcriptional inhibition of E-cadherin is triggered by some transcription factors. Snail is the first discovered transcription repressor of E-cadherin and has a critical function in this process 19. Surprisingly, we found that Snail was downregulated by miR-486-5p but upregulated by anti-miR-486-5p. Given that Dock1 may connect miR-486-5p and EMT, we investigated whether Dock1 connects miR-486-5p and Snail. Silence of Dock1 triggered a similar change in Snail expression compared with overexpression of miR-486-5p (Figure 4C). However, the alteration of Dock1 and miR-486-5p did not affect mRNA transcripts of Snail after 48 hours with or without 10 ng/mL of IL-22 (data not shown). As an inflammatory cytokine, IL-6 increases in a tumor environment, and is crucial to activate NF-κB pathway which can directly activate effective EMT inducers, such as Snail 20, 21. We first determined whether miR-486-5p and Dock1 were involved in Snail translocation in response to IL-22 stimulation. Our results manifested that the level of Snail was downregulated in the nuclear of SiDock1/MDA-MB-231 and miR-486-5p/MDA-MB-231 cells after 48 hours with 10 ng/mL IL-22, and restoration of Dock1 rescued the expression of Snail that was downregulated by miR-486-5p (Figure 4D). Then, we detected whether Dock1 and miR-486-5p modulate the stabilization of Snail via activation of NF-κB under stimulation of IL-22. Our data manifested that phosphorylation of IκBα was prominently downregulated in SiDock1/MDA-MB-231 and miR-486-5p/MDA-MB-231 cells after 48 hours with 10 ng/mL IL-22 and the restoration of Dock1 rescued the expression of IκBα phosphorylation that was inhibited by miR-486-5p (Figure 4E).

### MiR-486-5p is decreased and Dock1 is enhanced in breast cancer tissue, and the level of miR-486-5p and Dock1 is correlated with clinicopathological characteristics of breast cancer patients

**Figure 5:**
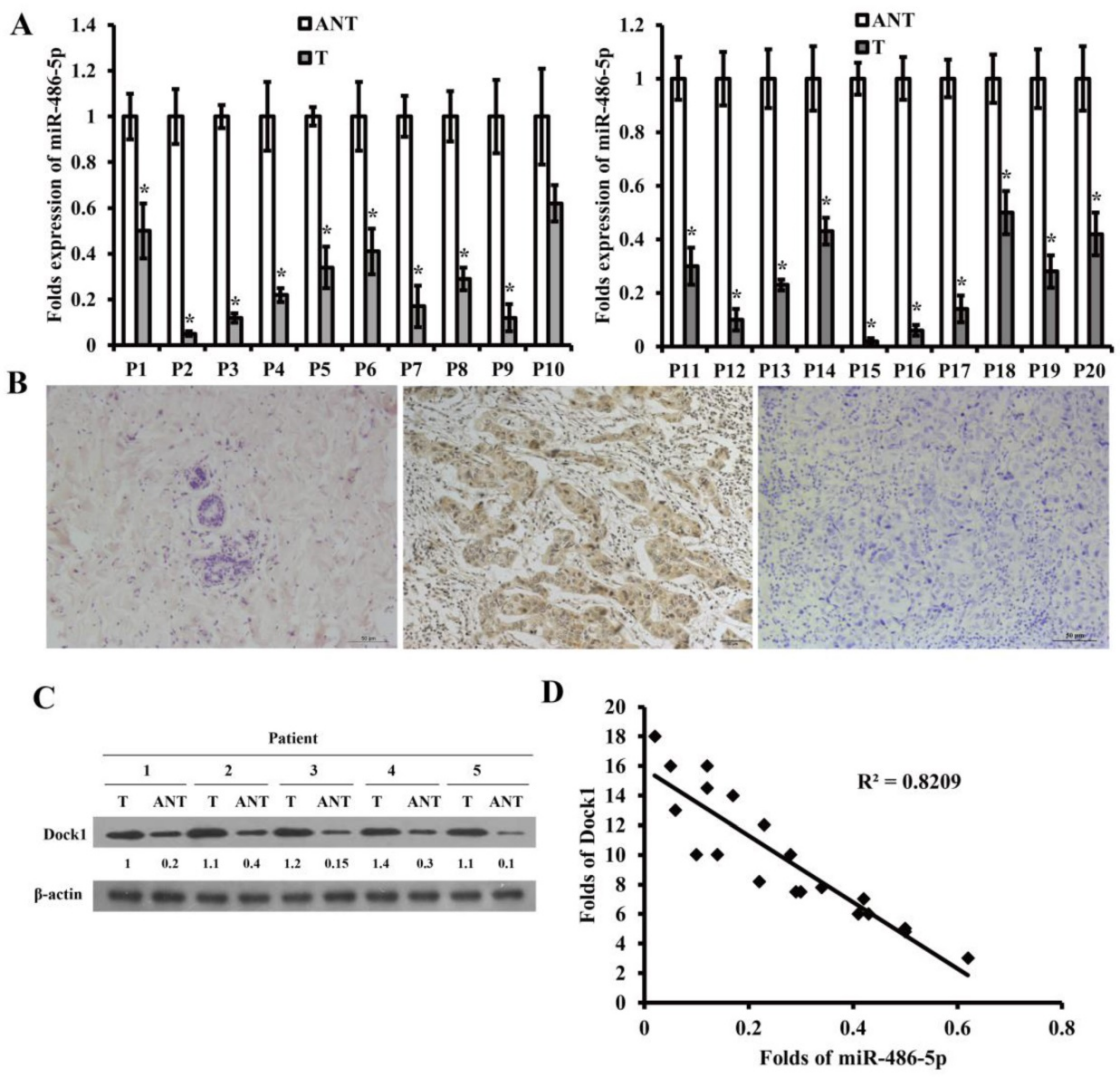
MiR-486-5p was downregulated in breast cancer and was correlated with patients' clinicopathological characteristics.** A.** Relative level of miR-486-5p in 20 pairs of frozen breast cancer tissues (T) and corresponding ANTs by qRT-PCR. **B.** The level of Dock1 in ANTs (a), breast cancer tissues with lymphatic and distant metastasis (b), and breast cancer tissues without metastasis (c), by immunohistochemistry. Scale bar: 50 μm. **C.** Western blot of the expression of Dock1 in breast cancer tissues (T) and ANTs. Used β-actin as a loading control. Relative protein level quantifications were shown below the blots. **D.** In human breast cancer tissues, the relevance between miR-486-5p and Dock1 expression.

To explore the potential function of miR-486-5p and Dock1 in genesis and progression of breast cancer, we detected the level of miR-486-5p using qRT-PCR and the level of Dock1 using immunohistochemistry and western blot in 80 pairs archived frozen breast cancer tissues and ANTs. The level of miR-486-5p was markedly decreased in most of the breast cancer tissues than ANTs (Figure 5A). The following results manifested that tumor differentiation, lymphatic and distant metastasis were associated with downregulation of miR-486-5p in breast cancer (Table 1), but not related to age, tumor size, or hormonal level (Table 1). Dock1 protein expression was markedly increased in most of the breast cancer tissues compared with corresponding ANTs (Figure 5B, C). The tumor differentiation, lymphatic and distant metastasis, and the levels of E-cadherin and Vimentin were correlated with upregulation of Dock1 in breast cancer (Table 1), but age, tumor size, or hormonal level were not (Table 1). In the same time, it manifested that the level of miR-486-5p was negatively associated with the level of Dock1 (illustrated by relative gray value) (Figure 5D).

### The invasion ability of breast cancer cells was reduced by downregulation of Dock1 and upregulation of miR-486-5p under stimulation with IL-22 *in vivo*

**Figure 6:**
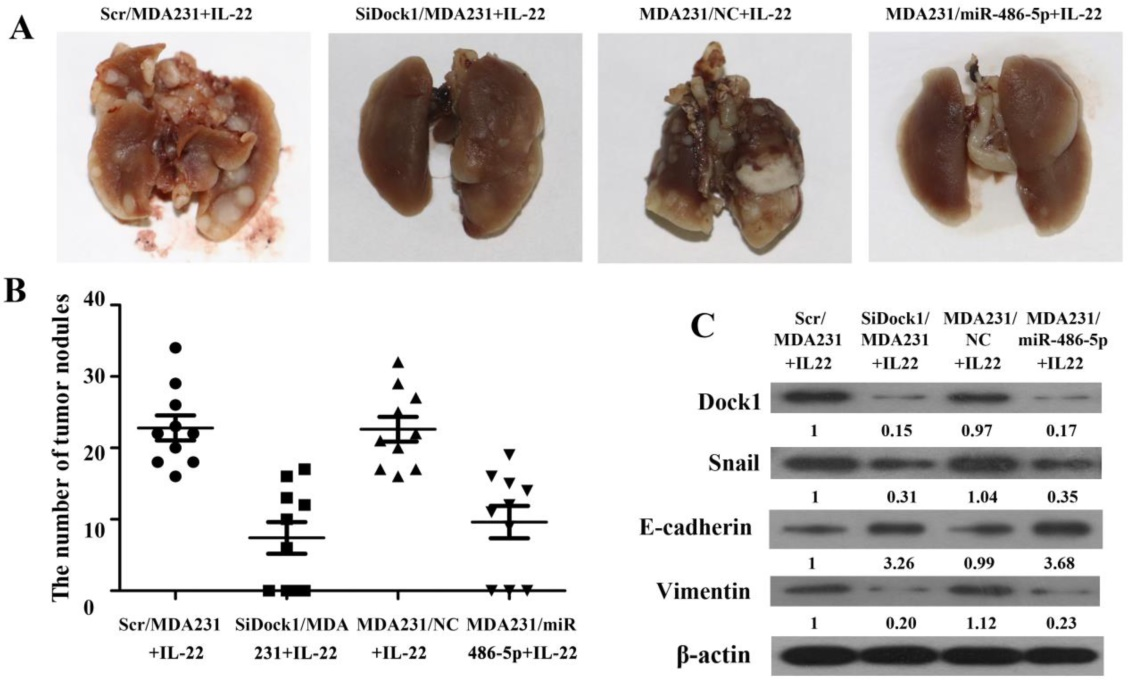
Reduction of Dock1 and upregulation of miR-486-5p reduced IL-22- induced breast cancer cell invasion *in vivo.* **A.** Comparison of lung metastasis between groups. **B.** Lung metastatic nodules were calculated and plotted (n=10).** C.** Expression of Dock1, Snail, E-cadherin, and Vimentin was determined in mice xenograft tumors by use of western blot. Used β-actin as a loading control. Quantifications of relative protein levels are shown below the blots.

The xenograft transplant model in SCID mice was established to assess the metastatic capability of breast cancer cells *in vivo*. As shown in Figure 6A and 6B, the pulmonary metastasis were counted after 4 weeks. The number of pulmonary metastasis nodules was reduced in the mice that was inoculated with SiDock1/MDA-MB-231 or miR-486-5p/MDA-MB-231 cells under stimulation with IL-22 compared with the group that was inoculated with Scr/MDA-MB-231 or NC/MDA-MB-231 cells. Moreover, the level of Dock1, Snail, and Vimentin in the tumor xenograft were downregulated, but E-cadherin level was upregulated in the mice that was inoculated with SiDock1/MDA-MB-231 or miR-486-5p/MDA-MB-231 cells than Scr/ MDA-MB-231 or NC/MDA-MB-231 cells (Figure 6C). These findings were coincident with results *in vitro*, indicating that miR-486-5p could inhibit IL-22-induced EMT through modulating Dock1.

## Discussion

Previous researches have denoted that IL-22, an important member of the IL-10 cytokine family, correlates with tumor growth and metastasis of many solid tumors^2^, ^3^, ^22^. IL-22 facilitated the invasion of gastric cancer cells by cancer-associated fibroblasts through ERK and STAT3 signaling 4. Consistent with previous findings, we also found that IL-22 promotes the metastasis and invasion of breast cancer cells. EMT plays vital role in tumor metastasis as a potential mechanism. Several oncogenic pathways, including TGF-β, PI3K, Wnt/β-catenin, MAPK, Hedgehog and Notch, have been confirmed to trigger EMT 23, 24. The transcription factors Snail and Slug suppress transcription of E-cadherin and facilitate EMT 25. Li et al. showed that Dock1 plays vital role in CXCL12-regulated metastasis and chemotaxis of breast cancer cells 26. Our previous study also showed that Dock1 acts vial part in the IL8-triggered EMT of glioma cells 6. This study found that Dock1 played important part on invasion, metastasis and EMT of breast cancer, which is consistent with previous findings.

In a number of human cancers, microRNAs (miRNA) play pivotal roles in tumor inhibition or carcinogenesis. Previous studies showed that miR-486-5p often expressed ectopically as an anti-oncogene in diverse cancers. For instance, miR-486-5p could act as an anti-oncogene and biomarker in non-small cell lung cancer (NSCLC), inhibiting the development and growth of NSCLC in mouse models 27, 28. The level of miR‑486 is low in osteosarcoma and patients who have low expression of miR-486 possess shorter median survival than those with higher miR‑486 29. Consistent with previous findings, it was discovered that miR-486-5p level was markedly reduced in breast cancer tissues than corresponding ANTs, thus miR-486-5p played as anti-oncogene in breast cancer. We discovered that the number of metastatic tumor nodules in lung of mice was lower in the group with overexpression of miR-486-5p or downregulation of Dock1 under stimulation with IL-22, suggesting miR-486-5p could repress breast cancer cells metastasis in mice models. The clinical features manifested that downregulation of miR-486-5p was associated with tumor differentiation, lymphatic and distant metastasis of breast cancer.

Previous studies have shown that miR-486 may suppress tumor progression, invasion, and migration. We found that miR-486-5p remarkably repressed invasion, metastasis, and EMT by binding and repressing Dock1. These findings were consistent with the results of several recent studies with other carcinoma cells. For example, the anti-oncogenic activity of miR-486 is probably related to targeting and suppressing OLFM4 in gastric cancer 30. Xiaoguang Zhang et al. found that prostate cancer metastasis is suppressed by miR-486-5p through binding Snail and inhibiting EMT 31. Our studies manifested that miR-486-5p suppressed the invasion, metastasis, and EMT of breast cancer cells.

A number of findings have manifested that NF-κB pathway is widely activated in diverse human cancer and is also significantly correlated with the development and metastasis of tumor 32, 33. Snail, a potent E-cadherin repressor, is a downstream target for NF-κB. A previous study showed that, miR-210-3p directly binds SOCS1 and TNIP1 through activating NF-κB pathway in prostate cancer cells, resulting in bone metastasis progression of prostate cancer 34. By downregulation of the NF-κB1-Snail1 pathway, miR-9 represses proliferation and metastasis of melanoma 35. Our results showed that miR-486-5p suppress IL-22-triggered EMT through repression of the Dock1/NF-κB/Snail pathway.

## Conclusions

In summary, our study demonstrated that IL-22 and Dock1 promoted the invasion, metastasis, and EMT of breast cancer cells. MiR-486-5p directly bound the Dock1 mRNA 3' untranslated region and inhibited IL-22-induced EMT of breast cancer cells via the Dock1/NF-κB/Snail signaling pathway. Dock1 overexpression reversed the effect caused by the overexpression of miR-486-5p.The current research offers new perception into metastasis and invasion of breast cancer cells and therefore may contribute to the progress of novel strategies against breast cancer.

## Figures and Tables

**Table 1:** Correlation of clinical features with miR-486-5p and Dock1 expression in breast cancer patients.

Variables	miR-486-5p expression		*p* Value	Dock1 expression		*p* Value
	High expression	Low expression		Positive expression	Negative expression	
**Age (years)**						
≤50	13	23	0.272	30	10	0.201
≥51	12	32		34	6	
**Tumor size (cm)**						
≤5cm	15	21	0.058	26	9	0.199
>5cm	10	34		38	7	
**Tumor differentiation**						
I	14	16	0.024	3	4	0.033
II	6	11		21	5	
III	5	28		40	7	
**Lymph node metastasis**						
Yes	9	37	0.009	25	12	0.010
No	16	18		39	4	
**Distant metastasis**						
Yes	10	35	0.042	31	13	0.017
No	15	20		33	3	
**ER**						
Positive	16	25	0.097	27	8	0.573
Negative	9	30		37	8	
**PR**						
Positive	17	27	0.091	29	6	0.392
Negative	8	28		35	10	
**c-erbB-2**						
Positive	11	28	0.371	40	7	0.141
Negative	14	27		24	9	
**E-cadherin**						
Positive	15	18	0.021	20	11	0.007
Negative	10	37		44	5	
**Vimentin**						
Positive	8	33	0.020	42	4	0.004
Negative	17	22		22	12	

## Abbreviations

miRNAs: microRNAs
EMT: Epithelial- mesenchymal transition
IL-22: Interleukin-22
Th17: T helper 17
DOCK1: Dedicator of cytokinesis 1
3'-UTR: 3'-untranslated regions
ANOVA: analysis of variance
ANTs: adjacent normal tissues
NSCLC: non-small cell lung cancer
FBS: fetal bovine serum
qRT-PCR: quantitative real-time PCR
CXCL12: C-X-C motif chemokine 12
OLFM4: Olfactomedin 4
SOCS1: suppressor of cytokine signaling 1
TNIP1: Tumor necrosis factor, alpha-induced protein 3-interacting protein 1
